# Twin Toughening‐Driven Martensitic Transformation Strategy Synergistic Improvement for Plasticity‐Thermal Shock Resistance of (Hf─Zr─Ti)C Ceramic Coating in Severe Thermal Environments

**DOI:** 10.1002/advs.202503226

**Published:** 2025-04-09

**Authors:** Jiachen Li, Yulei Zhang, Yanqin Fu, Tao Li, Jian Zhang, Deyu Yang, Lingfei Cao, Fanyu Lu, Junhao Zhao, Junshuai Lv, Hejun Li

**Affiliations:** ^1^ Shaanxi Key Laboratory of Fiber Reinforced Light Composite Materials Northwestern Polytechnical University Xi'an 710072 P. R. China; ^2^ Henan Key Laboratory of High Performance Carbon Fiber Reinforced Composites Institute of Carbon Matrix Composites Henan Academy of Sciences Zhengzhou 450046 P. R. China; ^3^ International Joint Laboratory for Light Alloys (Ministry of Education) College of Materials and Engineering Chongqing University Chongqing 400045 P. R. China

**Keywords:** martensitic twin transformation toughening, repeat ablation resistance, slip band‐twin transfer, stacking fault‐twin transfer, ultra‐high temperature ceramic coating

## Abstract

The inherent brittleness and insufficient thermal shock resistance of ultra‐high temperature ceramic (UHTC) in severe thermal environments (above 2000 °C) remain significant challenges. This characteristic notably shortens their operational lifespan as thermal protective coatings on structural composites in reusable aerospace applications. To address these challenges, a “ceramic self‐toughening strategy” is introduced, aimed at enhancing the plasticity and thermal shock resistance of (Hf─Zr─Ti)C coatings through twin toughening‐driven martensitic transformations in the oxide scale. In this work, the oxidation of (Hf_1/2_Zr_1/4_Ti_1/4_)C and (Hf_1/4_Zr_1/2_Ti_1/4_)C coatings produced Ti‐doped (Hf_2/3_Zr_1/3_)O_2_ and Ti‐doped (Hf_1/3_Zr_2/3_)O_2_, with martensitic transformations initiated by “slip band‐twin transfer” and “stacking fault‐twin transfer”, respectively. The mechanism facilitated the formation of stable, dense, and high‐toughness oxide scales after repeat ablation, and then endowed the prepared coatings with superior repeat ablation resistance than current thermal protective coatings. The findings elucidated the role of martensitic transformation mechanisms of Ti‐doped (Hf, Zr)O_2_ during repeat ablation, and provided general design guidelines for synergistically controlling the component, microstructure, toughness, and thermal shock resistance of UHTC blocks and UHTC‐modified composites in severe thermal environments.

## Graphical abstract

A “ceramic self‐toughening strategy” is introduced for enhancing the plasticity and thermal shock resistance of (Hf─Zr─Ti)C coatings through twin toughening‐driven martensitic transformations in oxide scales. It facilitated the formation of stable, dense, and high‐toughness oxide scales after repeat ablation, and endowed prepared coatings with superior repeat ablation resistance than current thermal protective coatings in severe thermal environments above 2000 °C.

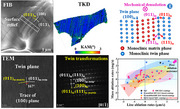

## 1 Introduction

Thermal structure components of reusable aerospace vehicles for space exploration and long‐distance transportation always suffer significant challenges during atmospheric reentry, where they are exposed to formidable thermal shock (temperatures exceeding 2000 °C) from high‐temperature oxidation, mechanical denudation, and temperature fluctuations associated with repeated ablation cycles.^[^ ^1^ ^]^ These extreme conditions necessitate advanced high‐temperature thermal structure materials with enhanced performance.^[^ ^2^, ^3^ ^]^ In recent years, ultra‐high temperature ceramic (UHTC)‐coated carbon/carbon (C/C) composites have drawn great attention due to benefiting from the high melting points (above 3000 °C) and good oxidation/ablation resistance of UHTC, as well as low density (≤2.0 g cm^−3^), high thermal conductivity and excellent strength retention rate of C/C composites at elevated temperatures.^[^ ^4^, ^5^, ^6^ ^]^ However, the inherent brittleness and insufficient thermal shock resistance of ceramics hinder their durability. The challenge lies in how to obtain stable, dense, and high‐toughness oxidation products for UHTC coatings in severe thermal environments that can resist formidable thermal shock damage during repeat ablation.^[^ ^7^, ^8^ ^]^

Various HfC/ZrC‐based coatings, designed in light of the high melting points of HfO_2_ (2810 °C) and ZrO_2_ (2670 °C), have been widely explored. Nevertheless, traditional monocarbide HfC and ZrC coatings developed loose coating surfaces under prolonged ablation due to the susceptibility of HfO_2_ and ZrO_2_ to mechanical denudation.^[^ ^9^, ^10^ ^]^ Our previous study demonstrated that the addition of TiC in HfC─ZrC coating densified the structure and improved the ablation resistance.^[^ ^11^ ^]^ Despite the enhancement, non‐uniform oxide product distribution (HfO_2_, ZrO_2_, and TiO_2_) after ablation hindered further improvements in resistance. Compared to HfC─ZrC─TiC, (Hf_1/3_Zr_1/3_Ti_1/3_)C medium‐entropy carbide offered a more homogeneous atomic‐scale compositional distribution, bringing the entropy stabilization effect and facilitating the synergistic oxidation among multi‐principal elements.^[^ ^12^ ^]^ This configuration promoted the formation of a stable and dense Ti‐doped (Hf, Zr)O_2_ oxide scale, as well as a decline in coating surface temperature via the volatilization of (Hf, Zr)TiO_4_, suppressing the mechanical denudation damage of oxides.^[^ ^10^, ^11^ ^]^ Nonetheless, ceramics’ brittleness, stemming from strong chemical bonds, imposed a high‐stress threshold against plastic deformation, impeding oxide scale toughness.^[^ ^13^, ^14^ ^]^ When (Hf_1/3_Zr_1/3_Ti_1/3_)C coating suffered the repeat ablation, the thermal shock damage was produced by high‐temperature oxidation, mechanical denudation, and temperature fluctuations during continuous ablative/cooling processes, triggering severe thermal stress concentration and then aggravating the rupture of the Ti‐doped (Hf, Zr)O_2_ oxide scale.

The development of high‐toughness oxide scales is crucial for overcoming the thermal shock limitations of UHTC coatings. Based on stress‐induced transformation models, Kelly et al.^[^ ^15^ ^]^ reviewed the effect of stress on crack initiation and propagation within ZrO_2_, and highlighted the potential of tetragonal‐to‐monoclinic phase transformation toughening within Zr‐based oxide scale for toughened applications. Actually, the tetragonal‐monoclinic phase transformation of ZrO_2_ was diffusionless and athermal transformation, which belonged to martensitic transformation in nature.^[^ ^16^ ^]^ Martensitic transformation also occurred in Hf─Zr‐based oxides (Hf‐rich Hf_x_Zr_1‐x_O_2_ and Zr‐rich Hf_x_Zr_1‐x_O_2_), attributed to the similar crystal structures, physical and chemical properties of HfO_2_ and ZrO_2_.^[^ ^17^ ^]^ Although the high melting points of Hf─Zr‐based oxides have shown great potential in the enhancement of ablation resistance, their martensitic transformation effect on the thermal shock resistance during repeat ablation has been grossly ignored.

Considering the martensitic transformation potential of Hf─Zr‐rich oxides and the sealing role of Ti‐rich oxides, we designed (Hf_1/2_Zr_1/4_Ti_1/4_)C (Hf─MEC) and (Hf_1/4_Zr_1/2_Ti_1/4_)C (Zr─MEC) coatings, by increasing the proportion of Hf/Zr contents in the Hf─Zr─Ti system. Their martensitic transformations, originating from twins, produced stable, dense, and high‐toughness oxide scales after repeat ablation, including Ti‐doped (Hf_2/3_Zr_1/3_)O_2_ in Hf─MEC coating and Ti‐doped (Hf_1/3_Zr_2/3_)O_2_ in Zr─MEC coating. The combination of FIB, TEM, and transmission Kikuchi diffraction (TKD) characterization revealed that the twin contributors for Ti‐doped (Hf_2/3_Zr_1/3_)O_2_ and Ti‐doped (Hf_1/3_Zr_2/3_)O_2_ were “slip band‐twin transfer” and “stacking fault‐twin transfer”, respectively. The twin toughening‐driven martensitic transformation strategy significantly enhances the plasticity‐thermal shock resistance of (Hf─Zr─Ti)C ceramic coating, extending their operational lifespan in severe thermal environments above 2000 °C compared to current thermal protective coatings.

## 2 Results and Discussion

### 2.1 Preparation and Repeat Ablation Resistance Test

**Figure 1:**
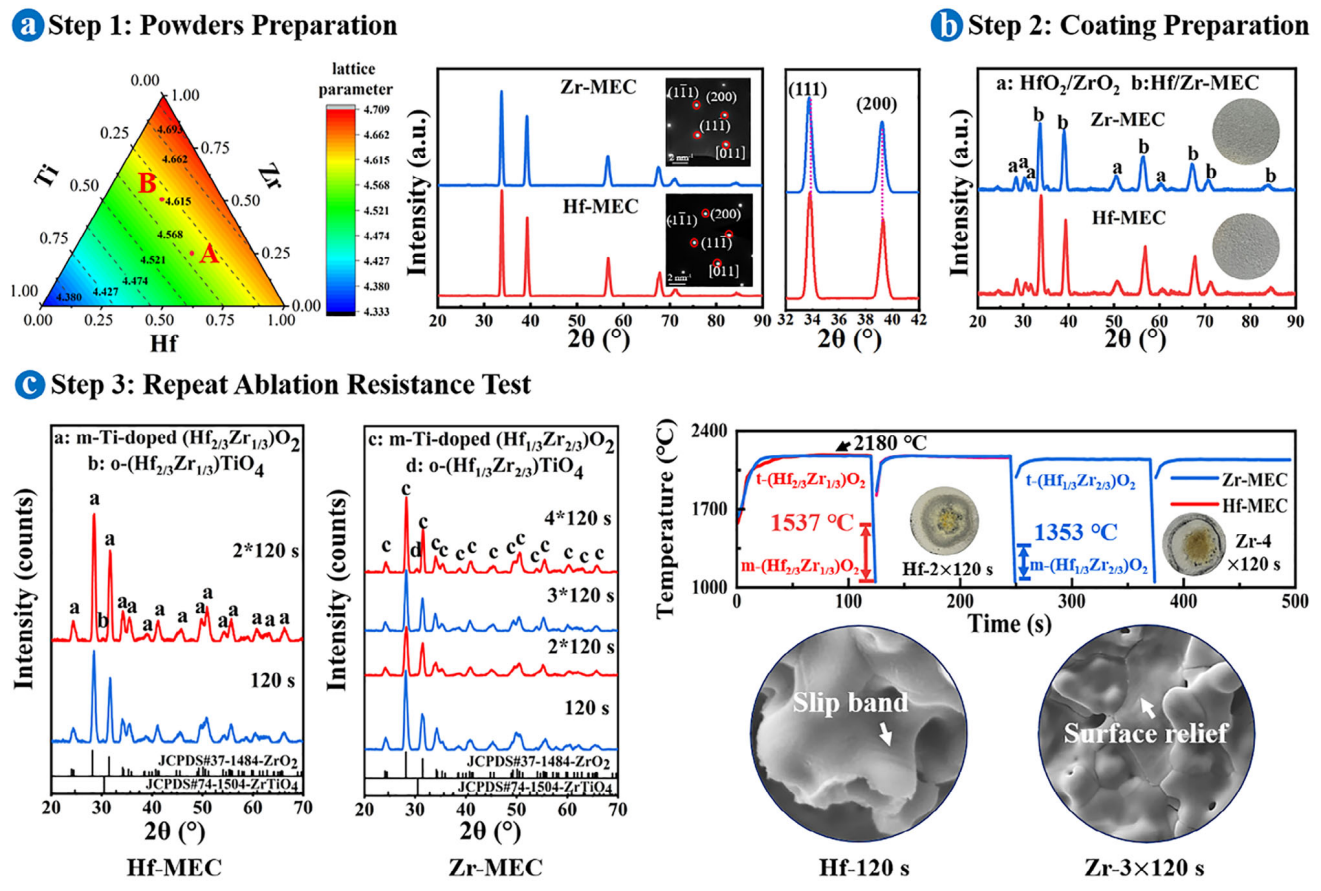
Preparation and repeat ablation resistance test of Hf/Zr─MEC‐coated C/C composites. a) Lattice parameters calculated by Vegard's law of all carbides in the Hf─Zr─Ti─C system and XRD patterns of Hf/Zr─MEC powders (inset SAED images). b) Macroscopic morphologies and XRD patterns of Hf/Zr─MEC‐coated C/C composites after SAPS. c) XRD patterns, temperature curves, and SEM morphologies of slip bands and surface reliefs (An ablation cycle included heating for 120 s and cooling for 5 s. The inset coatings in temperature curves represented the failed Hf/Zr─MEC coatings after ablation).

**Figure** **1** presents the preparation and repeat ablation resistance test of Hf/Zr─MEC‐coated C/C composites. Figure 1a shows the compositional and structural attributes of Hf/Zr─MEC powders (carbothermal reduction at 2100 °C). The lattice parameters of carbides within the Hf─Zr─Ti─C system were determined according to Vegard's law,^[^ ^18^ ^]^ expressed as,

(1) $$a=4.647x+4.709y+4.333\left(1-x-y\right)$$

where, *x* and *y* are the atomic percentages of the Hf and Zr atoms in the whole Hf─Zr─Ti system, respectively (both *x* and *y* increase in increments of 0.05 mol.%, ranging from 0 to 1), and the constants 4.647, 4.709, and 4.333 Å are lattice parameters of HfC, ZrC, and TiC,^[^ ^10^ ^]^ respectively. The calculated lattice parameters of Hf─MEC and Zr─MEC were 4.584 and 4.599 Å, respectively, which were in good agreement with the values of 4.585 and 4.597 Å from XRD.

Two independent groups of rock‐salt structure peaks indicated the formation of single‐phase Hf/Zr─MEC powders with an FCC structure, as further validated by the inset selective area electron diffraction (SAED) images. Due to the larger radius of the Zr atom compared to the Hf atom (*r*_Hf_ = 1.564 Å and *r*_Zr_ = 1.590 Å), the diffusion of Zr atoms to the Hf─Zr─Ti─C system promoted the lattice expansion (an increase in lattice parameter) and a decrease in 2θ. A smaller lattice expansion within Hf─MEC powders produced smaller interplanar spacing (0.265 nm, shown in HRTEM in Figure S1a, Supporting Information) compared to Zr─MEC powders (0.267 nm, Figure S1b, Supporting Information). The homogeneous distribution of multi‐components at the atomic level confirmed the successful preparation of Hf/Zr─MEC solid solution carbides through carbothermal reduction at 2100 °C.

After powder preparation, single‐phase Hf/Zr─MEC powders were granulated for fabricating Hf/Zr─MEC coatings on the surface of C/C composites. Figure 1b shows the XRD patterns and macroscopic morphologies of Hf/Zr─MEC coatings prepared by supersonic atmospheric plasma spraying (SAPS). The insufficiently melted particles induced particle‐stacking during SAPS, while melted particles hit the sample surface and then spread out to form planar areas (Figure S2a,b, Supporting Information), introducing localized oxides (Hf/ZrO_2_) on the coating surface (Figure 1b). The sprayed Hf/Zr─MEC coatings had comparable average thicknesses of ≈135 µm (Figure S2c,d, Supporting Information). Two sprayed coatings were intact without visible defects such as cracks or spallations, proving the successful preparation of Hf/Zr─MEC coatings by SAPS.

The repeat ablation resistance of Hf/Zr─MEC‐coated samples was tested by exposing them to an oxyacetylene flame with a heat flux of 2.4 MW m^−2^. After ablation, the main oxides of Hf/Zr─MEC coatings were Ti‐doped m‐(Hf_2/3_Zr_1/3_)O_2_ and Ti‐doped m‐(Hf_1/3_Zr_2/3_)O_2_, respectively, both of which belonged to Ti‐doped m‐(Hf, Zr)O_2_ (Figure 1c). The o‐(Hf_2/3_Zr_1/3_)TiO_4_ and o‐(Hf_1/3_Zr_2/3_)TiO_4_ appeared after 240 and 480 s of ablation, respectively, both corresponding to o‐(Hf, Zr)TiO_4_. Although Hf/Zr─MEC‐coated C/C composites had similar temperature paths, their oxide products underwent different phase transition behaviors. Both HfO_2_ and ZrO_2_ underwent cubic, tetragonal, and monoclinic transformations during the cooling process, with critical transition temperatures expressed by Equations (2) and (3),^[^ ^17^ ^]^

(2)

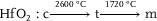



(3)

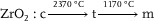




According to the molar ratio of oxides (neglecting the influence of a small amount of Ti doping on the transition temperatures), the transition temperatures of (Hf_2/3_Zr_1/3_)O_2_ and (Hf_1/3_Zr_2/3_)O_2_ were represented by the following equations,

(4)

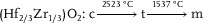



(5)

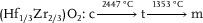




The tetragonal‐to‐monoclinic transformation of HfO_2_/ZrO_2_, driven by martensitic transformation, was independent of time, instead influenced by temperature variation.^[^ ^16^ ^]^ During each ablation cycle, coatings were heated to 2180 °C and then dropped to ≈1050 °C at each cooling gap. Since Ti‐doped (Hf_2/3_Zr_1/3_)O_2_ with a higher transformation temperature (1537 °C) than that of Ti‐doped (Hf_1/3_Zr_2/3_)O_2_ (1353 °C), underwent martensitic transformation earlier than that the latter during the cooling processes. It was confirmed by observing temperature‐induced early martensitic transformation in HfO_2_/Hf‐rich Hf_x_Zr_1‐x_O_2_ compared to ZrO_2_/Zr‐rich Hf_x_Zr_1‐x_O_2_, attributed to the former's greater nucleation driving force.^[^ ^17^ ^]^

The martensitic transformation initially released thermal shock‐induced stress, and delayed further accumulation, while subsequent ablation cycles generated stress that exceeded the stress tolerance of oxides, leading to local defect formation (cracks and pores) and eventual oxide rupture.^[^ ^13^, ^19^ ^]^ SEM analysis revealed that the emergence of the martensitic transformation coincided with plastic deformations (slip bands and surface reliefs) and crack formation. Figure S3 (Supporting Information) shows the detailed microstructural evolution of the Hf/Zr─MEC‐coated C/C composites after repeat ablation at different times. Notably, repeat ablation induced reverse tetragonal‐to‐monoclinic phase transformation upon reheating. Since m‐Ti‐doped (Hf_2/3_Zr_1/3_)O_2_ exhibited a wider martensitic transformation temperature range (1537–1050 °C) compared to m‐Ti‐doped (Hf_1/3_Zr_2/3_)O_2_ (1353–1050 °C), it experienced more martensitic transformations, leading to earlier occurrence of slip bands in Ti‐doped (Hf_2/3_Zr_1/3_)O_2_ after ablation for 120 s (Figure 1c; Figure S3a, Supporting Information), different from that of surface reliefs in Ti‐doped (Hf_1/3_Zr_2/3_)O_2_ after ablation for 360 s (Figure 1c; Figure S3d, Supporting Information). As ablation cycles increased, the continuous heating/cooling processes induced more rupture of the Ti‐doped (Hf_2/3_Zr_1/3_)O_2_ oxide scale, resulting in failures of the Hf─MEC coating after ablation for 240 s (Figure 1c; Figure S3b, Supporting Information) and the Zr─MEC coating after ablation for 480 s (Figure 1c; Figure S3e, Supporting Information), which demonstrated superior ablation resistance of Zr─MEC‐coated C/C composites. Additionally, due to the metastable nature of the tetragonal phase, its content was minimal or absent when cooling to room temperature after ablation.^[^ ^19^ ^]^

### 2.2 Synergistic Improvement of Plasticity and Thermal Shock Resistance

**Figure 2:**
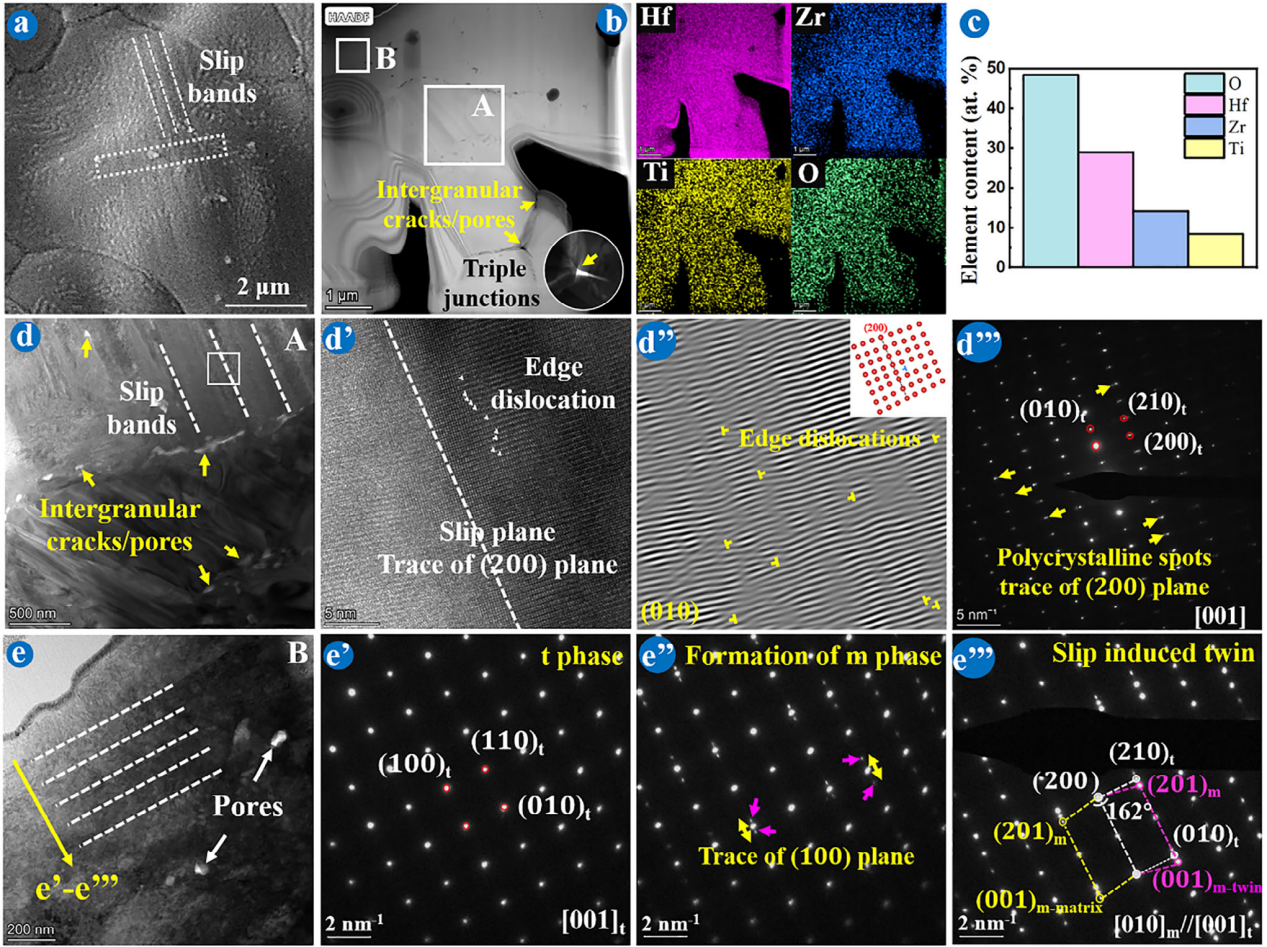
TEM images of FIB foils extracted from the Hf─MEC coating after ablation for 120 s. a) SEM of the sample selection area. b) HAADF image of the white box in a) and corresponding element mappings. c) EDS results of b). d‐e’’’) Bright field image, HRTEM, and SAED images in areas (d‐d’’’) A and (e‐e’’’) B (Schematic of an edge dislocation in inset (d’’)).

**Figure 3:**
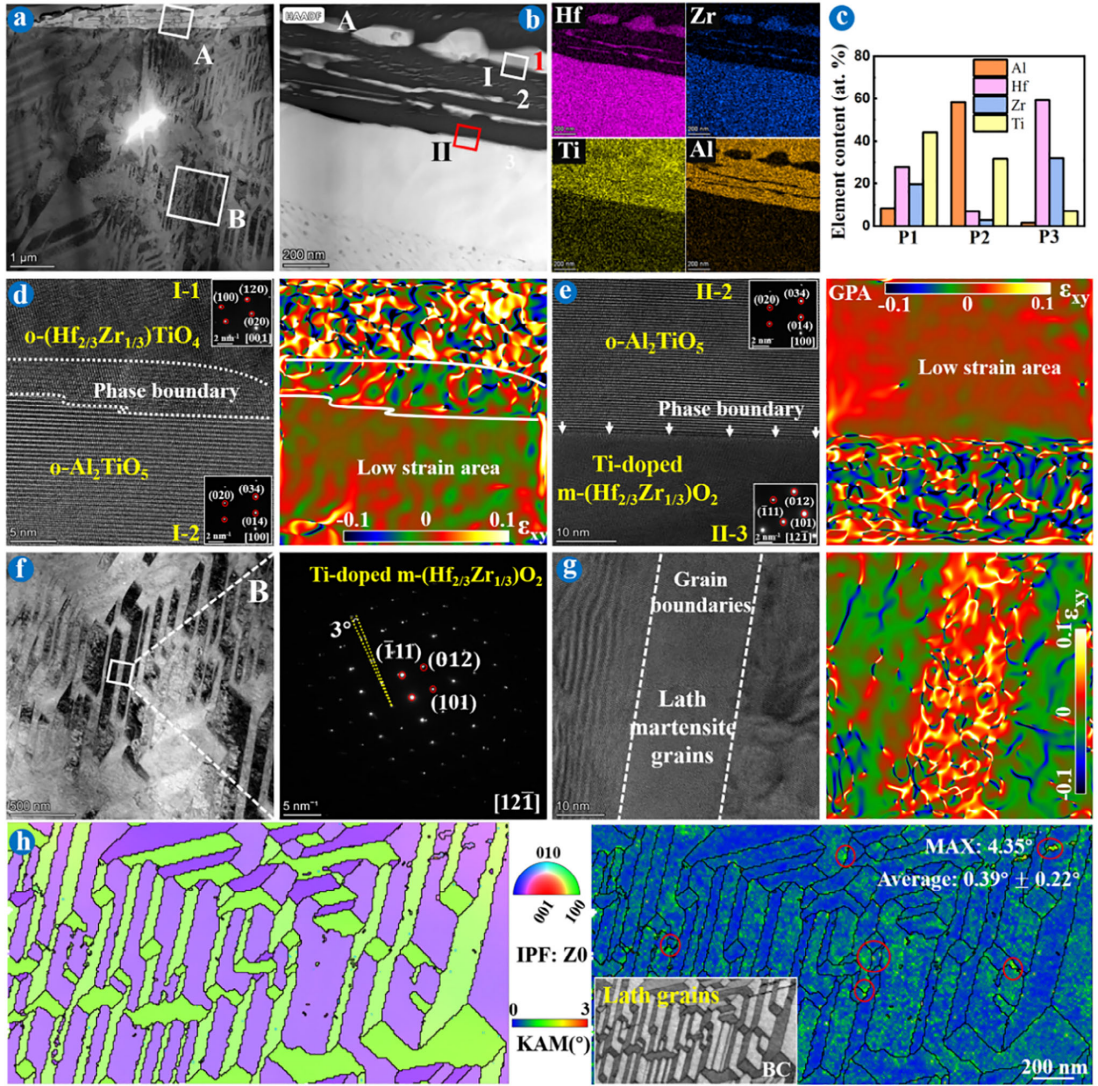
TEM images of a FIB extracted from the Hf─MEC coating after ablation for 2  × 120 s. a) HAADF of the sample selection area. b) HAADF image in area A and corresponding element mappings. c) EDS results. d,e) HRTEM (inset SAED figures) and related GPA images of the white box (d) I and (e) II in (b). f) HAADF image in area B and corresponding SAED. g) HRTEM and related GPA images of the white box in (f). h) Inverse pole figure (IPF), kernel average misorientation (KAM), and band contrast (BC) figures in area B.

**Figure 4:**
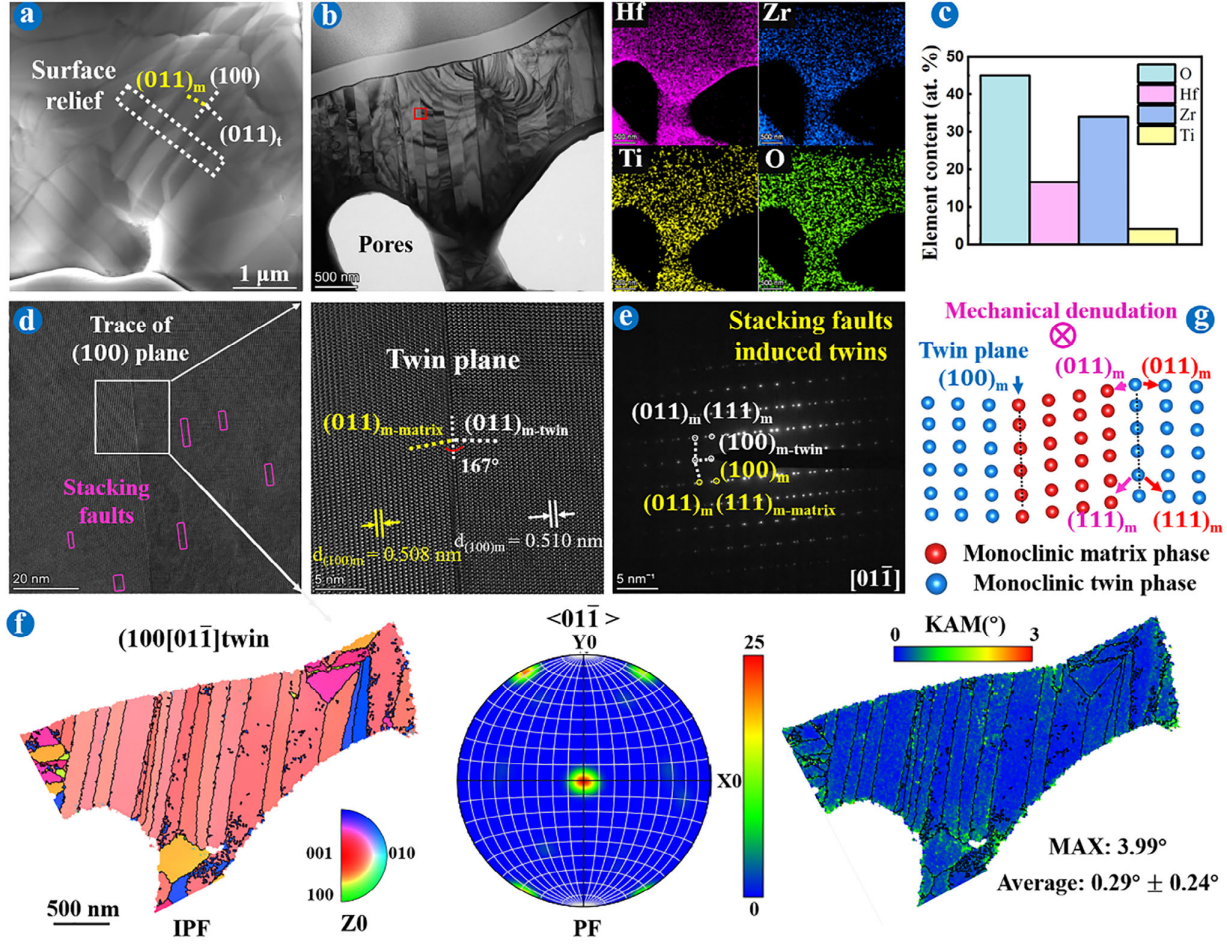
TEM images of FIB foils extracted from the Zr─MEC coating after ablation for 3  × 120 s. a) SEM of the sample selection area. b) Bright‐field image of the white box in (a) and corresponding element mappings. c–e) EDS result, HRTEM, and SAED images of the red box in (b). f) IPF, PF, and KAM figures in area B. g) Schematic of twins.

**Figure 5:**
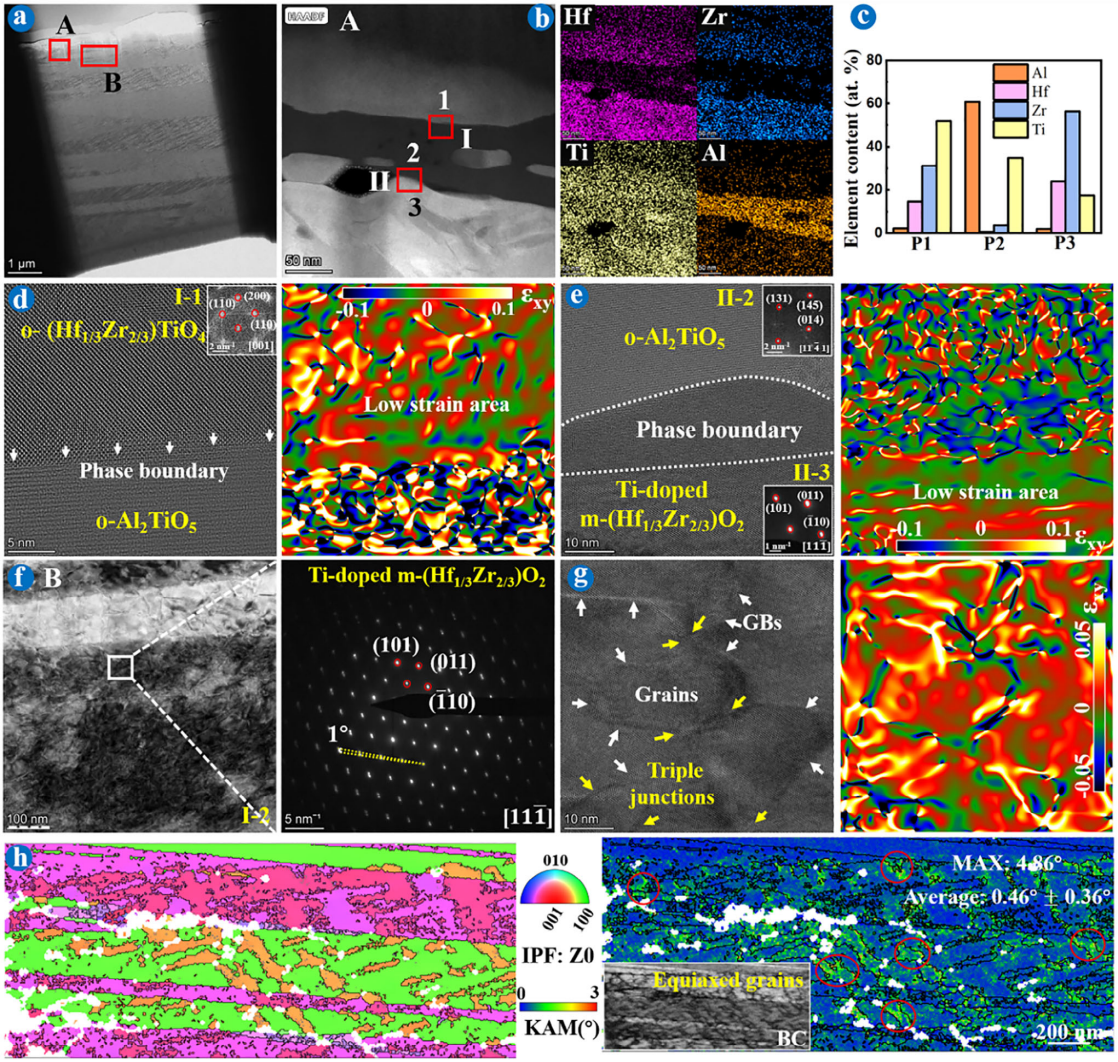
TEM images of a FIB extracted from the Zr─MEC coating after ablation for 4  × 120 s. a) HAADF of the sample selection area. b) HAADF image in area A and corresponding element mappings. c) EDS results. d,e) HRTEM (inset FFT and SAED figures) and related GPA images of the white box (d) I and (e) II in (b). f) HAADF image in area B and corresponding SAED. g) HRTEM and related GPA images of the red box in (f). h) IPF, KAM, and BC figures in area B.

TEM and TKD characterizations combining FIB technology were used to delve deeper into the underlying mechanisms of the martensitic transformation at the micro level, as depicted in Figures 2, 3, 4, 5. Analyses revealed that the Ti‐doped (Hf_2/3_Zr_1/3_)O_2_ and Ti‐doped (Hf_1/3_Zr_2/3_)O_2_ underwent distinct toughening mechanisms, classified as “slip band‐twin transfer” (**Figure** **2**) and “stacking fault‐twin transfer” (Figure 4) mechanisms, respectively.

Figure 2 is the TEM results of an extracted FIB foil from Hf─MEC coating subjected to 120 s of ablation. The elemental distribution and EDS results confirmed uniform composition across the Ti‐doped (Hf_2/3_Zr_1/3_)O_2_ oxide scale with a single‐component system during the martensitic transformation (Figure 2b,c).^[^ ^17^ ^]^ During initial ablation, thermal shock generated the stress within deformed grains. Since the martensitic transformation was a typical diffusionless transformation, the displacement of atoms was sub‐interatomic distance, without passing through adjacent grains.^[^ ^17^ ^]^ Stress was thereby released by plastic deformations (slip band, shear band twin, etc) instead of the atom migration.^[^ ^16^ ^]^ For the Ti‐doped (Hf_2/3_Zr_1/3_)O_2_ oxide scale, edge dislocation slip was the main toughening approach at the initial deformation. Plenty of edge dislocations slipped along the (200) plane in Figure 2d’ and 2d’’. The schematic of the edge dislocation is shown in the inset figure in Figure 2d’’. The continuous repeat ablation accelerated stress localization, which mobilized the accumulation of dislocations, followed by the formation of slip bands (Figure 2a,d).^[^ ^20^ ^]^

The exhaustion of dislocations at critical thresholds impeded the nucleation of additional dislocations for slip bands, triggering local ablative defect initiation. As a result, intergranular cracks/pores occurred around the triple junctions and slip bands (represented by transparent areas in Figure 2b,d). Conversely, new nucleating dislocations accumulated to the critical condition, gradually forming new deformed grains. The SAED image shows some diverged polycrystalline diffraction spots along the (200) plane (Figure 2d), confirmed by the same experimental phenomenon in area B (Figure 2e), along the direction of the yellow arrow. A set of diffraction spots for Ti‐doped t‐(Hf_2/3_Zr_1/3_)O_2_ first appeared (Figure 2e’), and then two sets of twin diffraction spots (m‐matrix and m‐twin^[^ ^21^ ^]^) were distributed along the (200) plane, changing from dark (Figure 2e’’) to bright (Figure 2e’’) with a twinning angle of 162°. The evidence for the martensitic transformation was found in the associated SAED pattern recorded along [010]_m_//[001]_t_^[^ ^15^ ^]^ (Figure 2e’’’). The accumulation of edge dislocation facilitated the transformation from slip bands to twins inside Ti‐doped (Hf_2/3_Zr_1/3_)O_2_ grains, thus slip bands being one of the contributors to the twin formation. Although the activation sequence of dislocation slip and twin might be altered depending on the local stress state or microstructures, the term “slip band‐twin transfer” mechanism was reasonable to elucidate the interacted behavior.^[^ ^22^, ^23^ ^]^

**Figure** **3** is the TEM and TKD results of an extracted FIB foil from Hf─MEC coating after ablation for 240 s, where the area was machined between the Ti‐poor and Ti‐rich phases boundary. The HAADF image and related elemental mappings revealed three distinct oxides, marked by points 1–3 in area A (Figure 3a–c). As ablative defects appeared on the coating surface, such as ablation pits (Figure S3b, Supporting Information) and intergranular cracks/pores (captured by transparent areas in Figure 2b inset), the SiC inner coating oxidized, resulting in gaseous SiO/SiO_2_ loss^[^ ^11^ ^]^ and subsequent outward diffusion of residual Al_2_O_3_, which reacted with Ti‐rich oxide to form Al_2_TiO_5_ at the boundary between o‐(Hf_2/3_Zr_1/3_)TiO_4_ (the upper phase, Figure 3d) and Ti‐doped m‐(Hf_2/3_Zr_1/3_)O_2_ (the bottom phase, Figure 3e). Calculated by geometric phase analysis (GPA), the in‐plane strain of Ti‐doped m‐(Hf_2/3_Zr_1/3_)O_2_ and o‐(Hf_2/3_Zr_1/3_)TiO_4_ were higher than that of Al_2_TiO_5_, implying localized strain concentration.^[^ ^24^ ^]^ The formation of Al_2_TiO_5_ repaired partial ablation defects and slowed down the rupture of the Ti‐doped (Hf_2/3_Zr_1/3_)O_2_ oxide scale. Additionally, Ti‐doped m‐(Hf_2/3_Zr_1/3_)O_2_ grains retained plenty of slip band structures, with lath grains of different orientations, particularly evident in area B in Figure 3f. The diffraction spots (captured from the triple‐grain junction) indicated that these lath grains exhibited a 3° angle difference, with high‐strain distribution in middle lath grains, as shown in Figure 3g. The lath structures and high‐strain accumulation were demonstrated by inverse pole figure (IPF), band contrast (BC), and kernel average misorientation (KAM) figures, as shown in Figure 3h, with a higher KAM reflecting a larger strain. Strains were distributed along grain boundaries (GBs), as presented by red circles, with a maximum misorientation of 4.35° and an average misorientation of 0.39° ± 0.22°. When stress was continuously accumulated, concentration strain caused the fracture of Ti‐doped m‐(Hf_2/3_Zr_1/3_)O_2_ grains, in the form of the lath shape.

**Figure** **4** analyzes the TEM results of an extracted FIB foil from Zr─MEC coating after ablation for 360 s. Unlike the slip bands at macroscopic and edge dislocations at microscopic within Hf─MEC coating, Zr─MEC coating represented macroscopic surface relief distribution (Figure 4a) attributed to the aggregation of microscopic twins (Figure 4b,e) and stacking faults (Figure 4d). In other words, unlike the “slip band‐twin transfer” mechanism for Ti‐doped m‐(Hf_2/3_Zr_1/3_)O_2_, Ti‐doped (Hf_1/3_Zr_2/3_)O_2_ underwent the “stacking fault‐twin transfer” mechanism. The elemental distribution also confirmed the Ti‐doped (Hf_1/3_Zr_2/3_)O_2_ oxide scale with a single‐component system (Figure 4b,c). Figure 4d is the high‐magnification image of the area marked by the red box in Figure 4b. The interplanar spacing of the left phase was 0.508 nm, and the interplanar spacing of the right phase was 0.510 nm, both belonged to the *d*_(100)_ of m‐HfO_2_ and m‐ZrO_2_, displaying a monoclinic structure (m‐matrix and m‐twin^[^ ^21^ ^]^). Their twin plane, along the (100) plane, connected two phases. The stacking faults along the same (100) plane play a role in the twin planes,^[^ ^25^ ^]^ providing further insight into the transformation process (Figure 4d). The corresponding SAED pattern shows a twin system along [01$\bar{1}$] zone axis with a twinning angle of 167° (Figure 4e), which was confirmed by the IPF and PF results with the [01$\bar{1}$] zone axis (Figure 4f), implying a (100)[01$\bar{1}$] twin system. Figure 4g is a schematic diagram of this twin system. Additionally, the KAM results showed no strain accumulation inside the twins, and the strains were mainly distributed along the edge of the pores below the sample, with a maximum misorientation of 3.99° and an average misorientation of 0.29° ± 0.24° (Figure 4f).

Similar to the TEM results of Hf─MEC coating after ablation for 240 s, Al_2_TiO_5_ also appeared at the interface between the Ti‐doped m‐(Hf_1/3_Zr_2/3_)O_2_ and o‐(Hf_1/3_Zr_2/3_)TiO_4_ for Zr─MEC coating after ablation for 480 s, as shown in **Figure** **5**. The formation of Al_2_TiO_5_ contributed to defect repair and stress relaxation. Interestingly, Al_2_TiO_5_ in Zr─MEC exhibited a higher strain than in Hf─MEC coating (Figure 5d,e), indicating lower stress localization in Ti‐doped m‐(Hf_1/3_Zr_2/3_)O_2_ and superior plasticity. Different from the lath grains within Ti‐doped m‐(Hf_2/3_Zr_1/3_)O_2_ (Figure 3f), equiaxed grains existed in Ti‐doped m‐(Hf_1/3_Zr_2/3_)O_2_ columnar grains (Figure 5f) with a 1° angle difference between these grains (captured from the white box). The high strain was mainly aggregated at the grain boundaries and triple junctions (Figure 3g,h, as presented by red circles,), as confirmed by the simulation of ZrO_2_ martensitic transformation under stress.^[^ ^26^, ^27^ ^]^ The KAM showed a maximum misorientation of 4.86° and an average strain of 0.46° ± 0.36°, higher than these in Figure 4f. Subgrain rotation gradually formed equiaxed grains (yellow areas in IPF figure) inside the columnar grains, followed by the grain boundaries rotation relaxing stress. This process was the main stress relaxation mechanism inside the equiaxed Ti‐doped m‐(Hf_1/3_Zr_2/3_)O_2_ grains.^[^ ^19^ ^]^ As the plasticity provided by the stacking faults hardly resisted stress localization, it caused the fracture of Ti‐doped m‐(Hf_1/3_Zr_2/3_)O_2_ grains, in the form of an equiaxed shape.

Figures 2, 3, 4, 5 showed the distinct fracture patterns in Ti‐doped m‐(Hf_2/3_Zr_1/3_)O_2_ and m‐(Hf_1/3_Zr_2/3_)O_2_ respectively, at the micro level during repeat ablation. Extreme thermal shock damage intensified the stress concentration, resulting in the rupture of lath Ti‐doped m‐(Hf_2/3_Zr_1/3_)O_2_ and equiaxed Ti‐doped m‐(Hf_1/3_Zr_2/3_)O_2_ grains, thus mainly appearing in the forms of cracks and pores at the macro level, respectively, as proved by the cross‐sectional BSE results of different coated samples after ablation in Figure S4 (Supporting Information). Notably, compared to the Hf─MEC coating, the cross‐sectional results showed the Zr─MEC coating exhibited superior repeat ablation resistance to thermal shock damage.

### 2.3 Twin Toughening‐Driven Martensitic Transformations

Calculated by the Scherrer model, the XRD results showed that the dislocation density of HfO_2_ (3.3  × 10^15^ m^−2^)^[^ ^28^ ^]^ exceeded that of ZrO_2_ (2.9  × 10^15^ m^−2^),^[^ ^29^ ^]^ which indicated HfO_2_ was prone to forming dislocations compared to ZrO_2_. The combination of SEM (Figure S3, Supporting Information) and TEM results (Figures 2, 3, 4, 5) revealed that the transformation mechanisms in Ti‐doped (Hf_2/3_Zr_1/3_)O_2_ and Ti‐doped (Hf_1/3_Zr_2/3_)O_2_ were triggered via the “slip band‐twin transfer” and “stacking fault‐twin transfer”, respectively.

**Figure 6:**
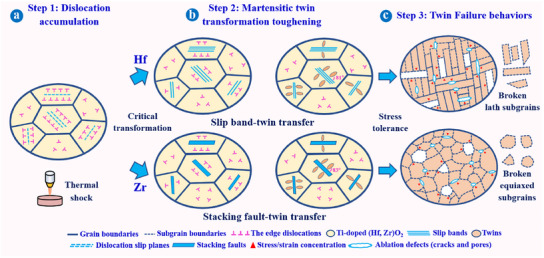
The schematic diagram of martensitic transformation toughening mechanisms and twin failure behaviors. a) Dislocation accumulation. b) Martensitic twin transformation toughening. c) Twin failure behaviors.

**Figure** **6** is the schematic illustration of martensitic transformation toughening mechanisms and twin failure behaviors in Ti‐doped (Hf, Zr)O_2_ within Hf/Zr─MEC coatings during repeat ablation. At the initial ablation, thermal shock damage produced stress and strain in Ti‐doped (Hf_2/3_Zr_1/3_)O_2_ (Hf─MEC coating) and Ti‐doped (Hf_1/3_Zr_2/3_)O_2_ (Zr─MEC coating). Not all the oxide grains can be triggered for martensitic transformation, and dislocation accumulation was their dominant stress relaxation approach (Figure 6a). When surpassing the critical stress and/or the crystal orientation of oxides was favorable for the transformation, the martensitic transformation was initiated (Figures 2, 6, and 4).^[^ ^30^ ^]^ The resulting toughening mechanisms (slip bands, stacking faults, and twins) were mutually constrained and coordinated with dislocations, which improved plasticity by increasing the storage capacity of dislocations to resist thermal shock damage during repeat ablation.^[^ ^30^ ^]^ However, once these mechanisms reached saturation, plastic deformation became challenging, leading to defect initiation (cracks and pores) and eventual failures (Figures 3, 6, and 5). Within twin grains, Ti‐doped (Hf_2/3_Zr_1/3_)O_2_ retained the lath subgrains of the slip bands, and the subgrain rotation for Ti‐doped (Hf_1/3_Zr_2/3_)O_2_ formed equiaxed grains. As a result, the stress concentration aggravated the rupture of Ti‐doped (Hf_2/3_Zr_1/3_)O_2_ and Ti‐doped (Hf_1/3_Zr_2/3_)O_2_, in the forms of lath and equiaxed grains at the micro level.

### 2.4 A Profile of Repeat Ablation Resistance

**Figure 7:**
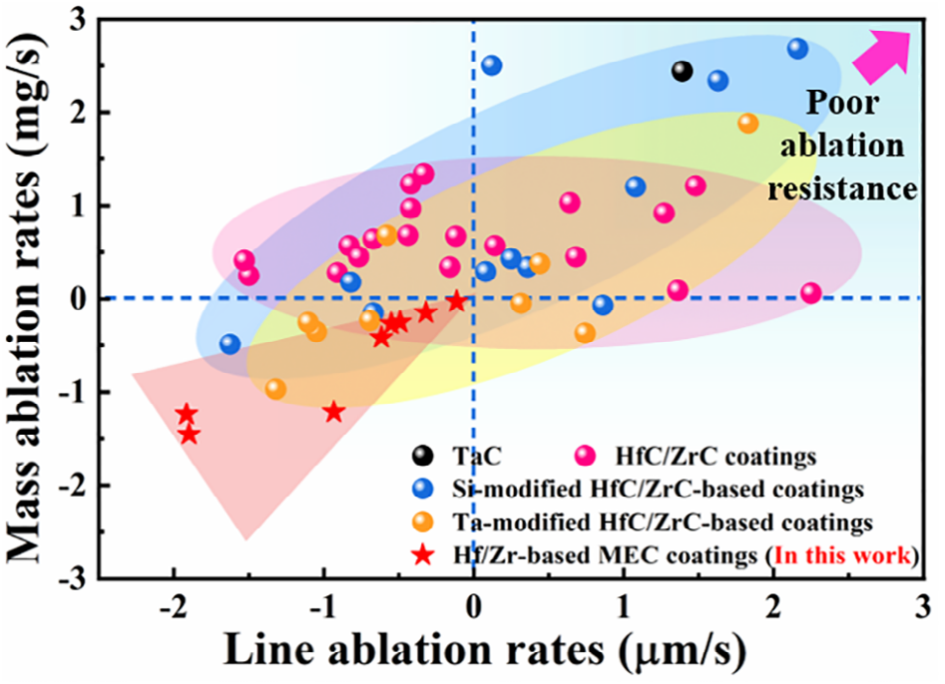
The comparison of oxyacetylene ablation resistance of different UHTC coatings at a heat flux of 2.4 MW/m^2^. Detailed information for different coatings is shown in Table S1 (Supporting Information).^[^ ^9^, ^32^, ^33^, ^34^, ^35^, ^36^, ^37^, ^38^, ^39^, ^40^, ^41^, ^42^, ^43^, ^44^, ^45^, ^46^ ^]^

**Figure** **7** presents a comparative analysis of different anti‐ablation coatings for C/C composites. The negative values indicate the presence of a good‐quality protective oxide layer on the coating surface, which resists the high‐speed airflow.^[^ ^2^ ^]^ Twin toughening‐driven martensitic transformation mechanism endowed Hf/Zr─MEC‐coatings with superior repeat ablation resistance than current thermal protective coatings. Traditional HfC/ZrC coatings displayed poor ablation resistance, as evidenced by positive mass and/or linear ablation rates (marked by the pink area). This limitation was attributed to their inability to form a protective barrier layer after prolonged ablation. In contrast, Si/Ta‐containing additives formed lower melting point oxides (SiO_2_ and Ta_2_O_5_) during ablation, followed by the solid solution reaction producing dense Hf/Zr─Si/Ta─O compound films. These compound layers healed the ablative defects (cracks and pores) and then improved the ablation resistance of HfC/ZrC coatings accordingly. Hence, the blue and yellow areas were positioned toward the bottom left corner, corresponding to the silicides/TaC‐modified HfC/ZrC coatings, respectively. However, the blue and yellow areas in the upper right corner implied a sharp increase in mass/line ablation rates because excessive SiO_2_ and Ta_2_O_5_ were prone to be taken away by mechanical denudation during ablation, leading to accelerated coating depletion. Especially, single‐phase TaC coating demonstrated high mass/line ablation rates, as indicated by a black sphere. Thus, Si/Ta‐containing additives were often used as secondary components to improve the ablation resistance of HfC/ZrC coatings. Due to the superior high‐temperature stability of Ta_2_O_5_ compared to SiO_2_, Hf/Zr─Ta─O compound films experienced less mass loss than Hf/Zr─Si─O compound films,^[^ ^31^ ^]^ The yellow area was located below the blue area.

In contrast, the twin toughening‐driven martensitic transformation mechanism of Ti‐doped (Hf, Zr)O_2_ synergistically improved the thermal shock resistance and service lifetime of the Hf/Zr─MEC‐coatings. Thereby, the mass/line ablation rates of the Hf/Zr─MEC coatings were located in the bottom left corner (marked by the red area). Compared to the ablation time of less than 180 s for the above anti‐ablation coatings, the Hf/Zr─MEC‐coated C/C composites were kept intact after ablation for 240 s/480 s, endowing their excellent resistance under repeated ablation conditions. Detailed information for different anti‐ablation coatings was provided in the supplementary document (Table S1, Supporting Information). A wider martensitic transformation temperature range of Ti‐doped (Hf_2/3_Zr_1/3_)O_2_ (1537–1050 °C, in Figure 1c), compared to Ti‐doped (Hf_1/3_Zr_2/3_)O_2_ (1353–1050 °C) during repeat ablation, promoted higher martensitic transformation contents of the former than the latter. It implied that a larger fraction of Ti‐doped (Hf_2/3_Zr_1/3_)O_2_ reached the stress tolerance than Ti‐doped (Hf_1/3_Zr_2/3_)O_2_ during an ablation cycle, thus aggravating strain concentration and failure on Ti‐doped (Hf_2/3_Zr_1/3_)O_2_ than Ti‐doped (Hf_1/3_Zr_2/3_)O_2_ caused by thermal shock damage. As a result, Zr─MEC coating exhibited superior repeat ablation resistance compared to Hf─MEC coating.

## 3 Conclusion

In summary, by integrating the martensitic transformation potential of Hf/Zr‐rich oxides and the sealing role of Ti‐rich oxides, we designed (Hf_1/2_Zr_1/4_Ti_1/4_)C (Hf‐MEC) and (Hf_1/4_Zr_1/2_Ti_1/4_)C (Zr─MEC) coatings applied in severe thermal environments above 2000 °C. FIB, TEM, and TKD characterizations revealed twin toughening‐driven martensitic transformation mechanisms and twin failure behaviors, further elucidating the effect on the plasticity‐thermal shock resistance of Hf/Zr─MEC coatings. The mechanisms were triggered via the “slip band‐twin transfer” and “stacking fault‐twin transfer” within Ti‐doped (Hf_2/3_Zr_1/3_)O_2_ and Ti‐doped (Hf_1/3_Zr_2/3_)O_2_, improving repeat ablation resistance. Thereby, Hf/Zr─MEC‐coated composites were kept intact after ablation for 2 × 120 s/4 × 120 s with the heating temperature up to 2180 °C, showing superior repeat ablation resistance than current thermal protective coatings. The proposed theories in this work offer valuable insights into the compositional optimization of UHTC blocks and UHTC‐modified composites, expanding the design space for excellent ablative composites.

## 4 Experimental Section

### Preparation

Commercial HfO_2_, ZrO_2,_ and TiO_2_ powders (all purities ≥99.0%, 1–3 µm) and graphite (≥99.9%, 1–3 µm) at molar ratios of 2:1:1:12 and 1:2:1:12 were ground for 6–8 h using a bench planetary mill. The mixed powders were heated by a heat treatment furnace at 2100 °C for 2–4 h under argon protection to obtain (Hf_1/2_Zr_1/4_Ti_1/4_)C (Hf─MEC) and (Hf_1/4_Zr_1/2_Ti_1/4_)C (Zr─MEC) powders.

(6) $$\begin{array}{ccc} 2\;\mathrm{Hf} & & O_{2}\left(s\right)+\mathrm{Zr}O_{2}\left(s\right)+\mathrm{Ti}O_{2}\left(s\right)+12C\left(s\right)\\ & & =4\left(Hf_{1/2}Zr_{1/4}Ti_{1/4}\right)C+8\mathrm{CO}\left(g\right) \end{array}$$

(7) $$\begin{array}{ccc} \mathrm{Hf} & & O_{2}\left(s\right)+2\mathrm{Zr}O_{2}\left(s\right)+\mathrm{Ti}O_{2}\left(s\right)+12C\left(s\right)\\ & & =4\left(Hf_{1/4}Zr_{1/2}Ti_{1/4}\right)C+8\mathrm{CO}\left(g\right) \end{array}$$

The gradient chemical vapor infiltration was carried out to deposit pyrolytic carbon into a 2.5D needle‐punched carbon fiber preform and obtain C/C composites with a density of ≈1.75 g cm^−3^. Commercial 10–20 wt.% graphite (300 mesh, ≥99.0%), 5–10 wt.% Al_2_O_3_ (300 mesh, ≥99.0%) and 70–80 wt.% Si (300 mesh, ≥99.0%) were mixed by a horizontal ball grinder for more than 6 h. After that, a graphite crucible containing these mixed powders and cylindrical C/C samples (size of Ф30 × 5 mm) was heated at a temperature range from 2000–2200 °C for 2–4 h within argon protection, fabricating the SiC‐coated C/C composites. Before preparing the Hf/Zr─MEC coatings, the synthesized Hf/Zr─MEC powders (20–50 wt.%) were mixed with distilled water (10–20 wt.%), ethanol (10–20 wt.%), and polyvinyl alcohol (20–50 wt.%) for 4–8 h, followed by a granulation through a spray dryer, with the inlet temperatures ranged from 320–350 °C and outlet temperatures were 110–140 °C. Then granulated powders were sprayed on the SiC‐coated C/C composites by SAPS, with 6–10 cycles, to obtain the Hf/Zr─MEC coatings. Based on previous explorations, the best spraying parameters and the preparation processes have been used to prepare the Hf─MEC and Zr─MEC coatings with comparable coating thicknesss.^[^ ^10^, ^11^ ^]^

t (45 kW) was used to prepare the Hf─MEC and Zr─MEC coatings with comparable coating thickness.

### Ablation Testing

An oxyacetylene torch (heat fluxes of 2.4 MW m^−2^) was used to test the repeat ablation resistance of the Hf/Zr─MEC‐coated C/C composites, of which the flame core temperature was up to 3000 °C. The flow rates of oxygen and acetylene were set as 0.24 and 0.18 L s^−1^, respectively. The distance between the flame gun nozzle and the surface of the specimen was 10 mm, and the surface center temperature was dynamically measured by a two‐color infrared thermometer (Raytek MR1SCSF). Each ablation cycle was 120 s and the samples were naturally cooled for 5 s within each ablation gap. The ablation resistance of different samples was determined by the mass (*R*_m_)/line (*R*_l_) ablation rates, which were the changes in mass/thickness between the before‐ablation and after‐ablation samples per unit time,

(8) $$R_{m}=\left(m_{0}-m_{1}\right)/\Delta t\;$$

(9) $$R_{l}=\left(l_{0}-l_{1}\right)/\Delta t\;$$

where *m*_0_ and *l*_0_ were the mass and thickness before ablation, while *m*_1_ and *l*_1_ were the mass and thickness after ablation, respectively. The Δ*t* is the ablation heating time.

### Characterization Techniques

The combination of X‐ray diffraction (XRD, X'Pert Pro MPD), scanning electron microscopes (SEM, JSM6460 and ZEISS EVO10) and transmission electron microscopes (TEM, Themis Z) with equipped energy dispersion spectroscopy (EDS) was used to study the compositional and structural information of Hf/Zr─MEC powders synthesized by carbothermal reduction and Hf/Zr─MEC‐coated C/C composites through SAPS. After ablation, the ablated Hf/Zr─MEC‐coated samples were machined by the thinning method and focused ion beam (FIB, Helios G4 CX) microscopy to obtain the FIB film, followed by TEM and TKD characterization. The compositional and structural information was analyzed by selective area electron diffraction (SAED), high‐resolution transmission electron microscopy (HRTEM), and fast Fourier transformation (FFT, obtained by Digital Micrograph software). In addition, the geometric phase analysis (GPA) was carried out to calculate the strain fields within HRTEM images through a plug‐in GPA software for the image‐processing package in Digital Micrograph software. TKD characterization was carried out by Tescan Clara GMH and all the TKD maps were further cleaned using the AZTEC software.

## Conflict of Interest

The authors declare no conflict of interest.

## Author Contributions

J.C.L., Y.L.Z., and H.J.L. conceived the idea. J.C.L. prepared the materials, performed the tests, conducted the characterization, and wrote the manuscript. Y.L.Z. supervised the research. Y.Q.F., T.L., D.Y.Y., and L.F.C. contributed to the data analysis. J.Z., F.Y.L., J.H.Z., and J.S.L. assisted in the experiments. All authors contributed to the discussion of the results and commented on the manuscript.

## Supporting information



Supporting Information

## Data Availability

All data needed to evaluate the conclusions are present in the paper and the Supplementary Materials. Source data are provided in this paper.

## References

[1] M. Yan , C. Hu , J. Li , R. Zhao , S. Pang , B. Liang , S. Tang , G. Liu , H. M. Cheng , Adv. Funct. Mater. 2022, 32, 2204133.

[2] Y. Zeng , D. Wang , X. Xiong , X. Zhang , P. J. Withers , W. Sun , M. Smith , M. Bai , P. Xiao , Nat. Commun. 2017, 8, 15836. 28613275 10.1038/ncomms15836 PMC5474735

[3] J. Lv , W. Li , Y. Fu , M. Zhang , L. Guo , F. Lu , J. Li , T. Li , Y. Zhang , H. Li , Adv. Sci. 2025, 12, 2411292. 10.1002/advs.202411292 PMC11904937 39812219

[4] L. Guo , S. Huang , W. Li , J. Lv , J. Sun , Adv. Powder Mater. 2024, 3, 100213.

[5] Z. Wen , Z. Tang , Y. Liu , L. Zhuang , H. Yu , Y. Chu , Adv. Mater. 2024, 36, 2311870. 10.1002/adma.202311870 38166175

[6] J. Lv , W. Li , T. Li , B. Gao , J. Li , Y. Fu , L. Guo , Y. Zhang , J. Mater. Sci. Technol. 2025, 204, 115.

[7] J. Li , J. Zhao , T. Li , J. Li , D. Yang , Y. Fu , J. Lv , L. Guo , Y. Zhang , Compos. Part B Eng. 2024, 281, 111569.

[8] M. Rühle , A. G. Evans , Prog. Mater. Sci. 1989, 33, 85.

[9] J. Li , T. Li , C. Huang , D. Yang , J. Lv , R. Zhang , Y. Jiao , Y. Zhang , Corros. Sci. 2024, 228, 111795.

[10] J. Li , Y. Zhang , Y. Zhao , Y. Zou , J. Lv , J. Li , Compos. Part B Eng. 2023, 251, 110467.

[11] J. Li , Y. Zhang , J. Lv , T. Li , X. Zhu , W. Gai , Corros. Sci. 2022, 205, 110474.

[12] J. Li , F. Lu , T. Li , Y. Fu , J. Zhao , J. Lv , Y. Zhang , J. Adv. Ceram. 2024, 13, 1223.

[13] L. R. Dong , J. Zhang , Y. Z. Li , Y. X. Gao , M. Wang , M. X. Huang , J. S. Wang , K. X. Chen , Science 2024, 385, 422. 39052815 10.1126/science.adp0559

[14] J. Zhang , G. Liu , W. Cui , Y. Ge , S. Du , Y. Gao , Y. Zhang , F. Li , Z. Chen , S. Du , K. Chen , Science 2022, 378, 371. 36302007 10.1126/science.abq7490

[15] P. M. Kelly , L. R. F. Rose , Prog. Mater. Sci. 2002, 477, 463.

[16] G. M. Wolten , J. Am. Ceram. Soc. 1963, 46, 418.

[17] J. Tang , F. Zhang , P. Zoogman , J. Fabbri , S. W. Chan , Y. Zhu , L. E. Brus , M. L. Steigerwald , Adv. Funct. Mater. 2005, 15, 1595.

[18] L. Chen , W. Zhang , Y. Tan , P. Jia , C. Xu , Y. Wang , X. Zhang , J. Han , Y. Zhou , J. Eur. Ceram. Soc. 2021, 41, 60.

[19] J. Liu , Y. Xie , Z. Hao , Z. Cui , R. Meng , F. Zhao , J. Yang , W. Liu , S. Xie , P. Hu , G. Bai , D. Yun , Acta Mater. 2023, 256, 119114.

[20] C. Sun , H. Wu , W. Chi , W. Wang , G. Zhang , Int. J. Fatigue. 2023, 167, 107331.

[21] J. Hu , Q. Yang , S. Zhu , Y. Zhang , D. Yan , K. Gan , Z. Li , Nat. Commun. 2023, 14, 5717. 37714826 10.1038/s41467-023-41481-6 PMC10504279

[22] S. Wei , G. Zhu , C. C. Tasan , Acta Mater. 2021, 206, 116520.

[23] J. Yang , W. Wang , M. Zhang , L. Chen , H. Zhang , Mater. Today Commun. 2024, 39, 109031.

[24] S. Y. Wang , F. C. Lang , Y. M. Xing , Compos. Sci. Technol. 2023, 242, 110215.

[25] M. V. Pantawane , S. Sharma , A. Sharma , S. Dasari , S. Banerjee , R. Banerjee , N. B. Dahotre , Acta Mater. 2021, 213, 116954.

[26] E. Camposilvan , M. Anglada , Acta Mater. 2016, 103, 882.

[27] M. Mamivand , M. Asle Zaeem , H. El Kadiri , Int. J. Plasticity. 2014, 60, 71.

[28] P. Harishsenthil , J. Chandrasekaran , R. Marnadu , V. Balasubramani , Sensor. Actuat. A‐Phys. 2021, 331, 112725.

[29] O. Aldaghri , B. A. El‐Badry , K. H. Ibnaouf , K. K. Taha , M. A. B. Aissa , A. Modwi , Diam. Relat. Mater. 2024, 144, 110944.

[30] W. Zheng , Y. Shi , L. Zhao , S. Jia , L. Li , C. L. Gan , D. Zhang , Q. Guo , Nat. Commun. 2023, 14, 7103. 37925460 10.1038/s41467-023-42815-0 PMC10625574

[31] Y. Jiang , C. Hu , B. Liang , S. Pang , J. Li , S. Tang , Sur. Coat. Tech. 2022, 451, 129072.

[32] J. Kong , Y. Zhang , G. Chen , P. Zhang , W. Gai , H. Wang , H. Li , J. Eur. Ceram. Soc. 2022, 42, 6898.

[33] J. Ren , Y. Zhang , P. Zhang , T. Li , J. Li , Y. Yang , J. Eur. Ceram. Soc. 2017, 37, 2759.

[34] J. Zhang , Y. Zhang , Y. Fu , T. Li , J. Meng , Vacuum 2019, 169, 108886.

[35] J. Zhang , Y. Zhang , Y. Fu , Y. Zhang , X. Zhu , Corros. Sci. 2021, 192, 109819.

[36] J. Ren , Y. Zhang , J. Zhang , Y. Fu , S. Tian , Ceram. Int. 2018, 44, 11340.

[37] J. Zhang , Y. Zhang , Y. Fu , X. Zhu , R. Chen , Corros. Sci. 2021, 189, 109586.

[38] Y. Jia , H. Li , Q. Fu , Z. Zhao , J. Sun , Corros. Sci. 2017, 123, 40.

[39] Y. Jia , H. Li , J. Sun , L. Li , Q. Fu , Int. J. Appl. Ceram. Tec. 2017, 14, 331.

[40] J. Ren , E. Feng , Y. Zhang , J. Zhang , D. Ding , L. Li , Ceram. Int. 2021, 47, 556.

[41] J. Ren , Y. Zhang , Y. Fu , P. Zhang , S. Tian , L. Zhang , Sur. Coat. Tech. 2018, 344, 250.

[42] Y. Wang , H. Li , Q. Fu , H. Wu , D. Yao , H. Li , Sur. Coat. Tech. 2012, 206, 3883.

[43] J. Zhang , Y. Zhang , Y. Fu , T. Zhang , X. Zhu , Corros. Sci. 2021, 192, 109853.

[44] K. Shuai , Y. Zhang , Y. Fu , X. Guo , T. Li , J. Li , Corros. Sci. 2021, 193, 109884.

[45] B. Li , H. J. Li , X. Y. Yao , X. F. Tian , Y. J. Jia , G. H. Feng , J. Mater. Sci. Technol. 2022, 115, 129.

[46] G. Feng , Y. Yu , X. Yao , Y. Jia , J. Sun , H. Li , J. Eur. Ceram. Soc. 2022, 42, 830.

[47] G. Feng , H. Li , X. Yao , M. Chen , Y. Xue , Ceram. Int. 2019, 45, 17936.

[48] J. Zhang , Y. Zhang , X. Zhu , Y. Fu , R. Chen , W. Gai , Corros. Sci. 2022, 204, 110385.

[49] G. Feng , H. Li , L. Yang , B. Li , J. Xu , X. Yao , Corros. Sci. 2020, 170, 108649.

[50] J. Ren , E. Feng , Y. Zhang , J. Zhang , L. Li , Ceram. Int. 2020, 46, 10147.

[51] G. Feng , H. Li , X. Yao , H. Zhou , Y. Yu , J. Lu , J. Eur. Ceram. Soc. 2021, 41, 3207.

