# Supplementary material for: Twin Toughening‐Driven Martensitic Transformation Strategy Synergistic Improvement for Plasticity‐Thermal Shock Resistance of (Hf─Zr─Ti)C Ceramic Coating in Severe Thermal Environments
Source: Adv Sci (Weinh). 2025 Apr 9;12(26):2503226. doi: 10.1002/advs.202503226 (PMC12245112; doi:10.1002/advs.202503226)
Supplement: Supplementary file 1 — Supporting Information [file ADVS-12-2503226-s001.docx]

**Supplementary Materials**

**Twin toughening-driven martensitic transformation strategy synergistic improvement for plasticity-thermal shock resistance of (Hf-Zr-Ti)C ceramic coating in severe thermal environments**

Jiachen Li ^1^, Yulei Zhang ^1,2^*, Yanqin Fu ^2^, Tao Li ^2^, Jian Zhang ^1^, Deyu Yang ^1^, Lingfei Cao ^3^, Fanyu Lu ^1^, Junhao Zhao ^1^, Junshuai Lv ^1^, Hejun Li ^1^*

*^1^ Shaanxi Key Laboratory of Fiber Reinforced Light Composite Materials, Northwestern Polytechnical University, Xi’an 710072, PR China*

^2^ *Henan Key Laboratory of High Performance Carbon Fiber Reinforced Composites, Institute of Carbon Matrix Composites, Henan Academy of Sciences, Zhengzhou 450046, China*

^3^ *International Joint Laboratory for Light Alloys (Ministry of Education), College of Materials and Engineering, Chongqing University, Chongqing 400045, China*

^*^*Corresponding author.* [zhangyulei@nwpu.edu.cn](mailto:zhangyulei@nwpu.edu.cn); [lihejun@nwpu.edu.cn](mailto:lihejun@nwpu.edu.cn)

***Keywords:*** Ultra-high temperature ceramic coating; Martensitic twin transformation toughening; Slip band-twin transfer; Stacking fault-twin transfer; Repeat ablation resistance


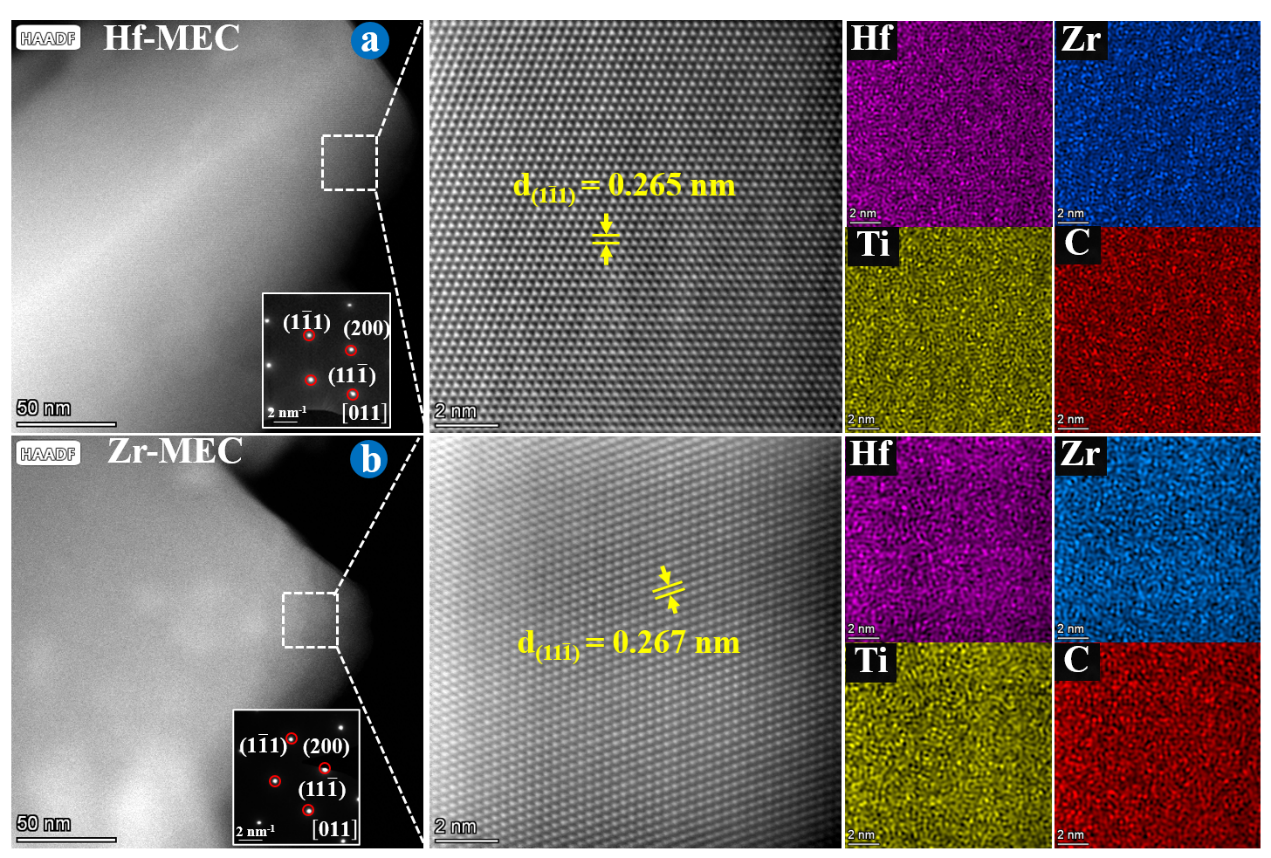


**Figure S1.** Preparation of Hf/Zr-MEC powders. The HAADF, SAED, HRTEM and corresponding element mappings of a) Hf-MEC and b) Zr-MEC powders.


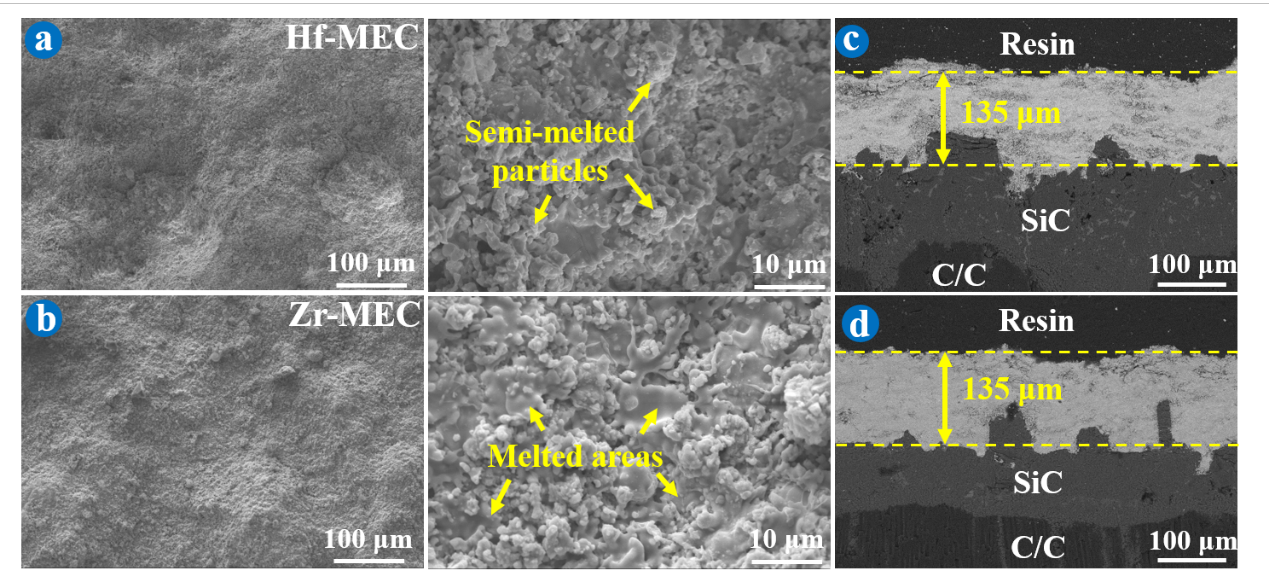


**Figure S2.** Preparation of Hf/Zr-MEC-coated C/C composites. a, b) Surface SEM and c, d) Cross-sectional BSE images of a, c) Hf-MEC-coated and b, d) Zr-MEC-coated C/C composites after SAPS.

**Surface compositional and structural attributes of Hf/Zr-MEC-coated C/C composites.**

Figure S3 shows the macroscopic morphologies and SEM results of the Hf/Zr-MEC-coated C/C composites after repeat ablation at different times. For the Hf-MEC coating, a crack appeared on the surface of the Ti-doped (Hf_2/3_Zr_1/3_)O_2_ oxide scale after ablation for 120 s (marked by P1 in Figures S3a). The slip bands (one of the plastic deformations) occurred around the crack, meaning the strain tolerance of the partial oxide grains to the critical value under mechanical denudation. Thus, as the ablation time increased to 240 s, the repeat ablation induced more severe stress concentration, which aggravated the rupture of these oxide scales and then formed an ablation pit, as shown in Figure S3b. Oxygen permeated these ablative defects inward until the SiC inner coating. The active oxidation of SiC in a low oxygen partial pressure environment produced gaseous SiO, and the passive oxidation generated gaseous SiO_2_ in an oxygen-rich condition. Both two kinds of gaseous accelerated the consumption of SiC and reduced thermal protection ability. Additionally, the remaining Al_2_O_3_ in the SiC inner coating diffused outward and reacted with Ti-rich oxide, forming Al_2_Ti_7_O_15_ (P2 in Figure S3b), which repaired partial ablation defects and slowed down the damage of Hf-MEC-coated C/C composites.

Due to the wider martensitic transformation temperature range of Ti-doped (Hf_2/3_Zr_1/3_)O_2_ than that of Ti-doped (Hf_1/3_Zr_2/3_)O_2_, the latter reached the critical strain tolerance later than that of the former. Therefore, no plastic deformations were found on the surface of the Ti-doped (Hf_1/3_Zr_2/3_)O_2_ oxide scale (marked by P4 in Figures S3c) for Zr-MEC coating after ablation for 240 s. As the ablation time increased to 360 s, surface reliefs appeared around microcracks under mechanical denudation (Figures S3d), which was another plastic deformation different from the slip bands in Ti-doped (Hf_2/3_Zr_1/3_)O_2_. Similar to the failure of the Hf-MEC coating after ablation for 240 s, the existence of Al_2_TiO_5_ implied the damage of the Zr-MEC coating after ablation for 480 s (Figures S3e). When Ti-doped (Hf_2/3_Zr_1/3_)O_2_ (Hf-MEC coating) and Ti-doped (Hf_1/3_Zr_2/3_)O_2_ (Zr-MEC coating) were subjected to formidable thermal shock damage during repeat ablation, the stress release at the macro level was mainly depended on slip bands and surface reliefs, respectively. Compared to the occurrence of surface reliefs in Ti-doped (Hf_1/3_Zr_2/3_)O_2_ after ablation (Figure S3d) for 360 s, the premature occurrence of slip bands in Ti-doped (Hf_2/3_Zr_1/3_)O_2_ after ablation for 120 s (Figure S3a) indicated the better ablation resistance of the former than that of the latter.


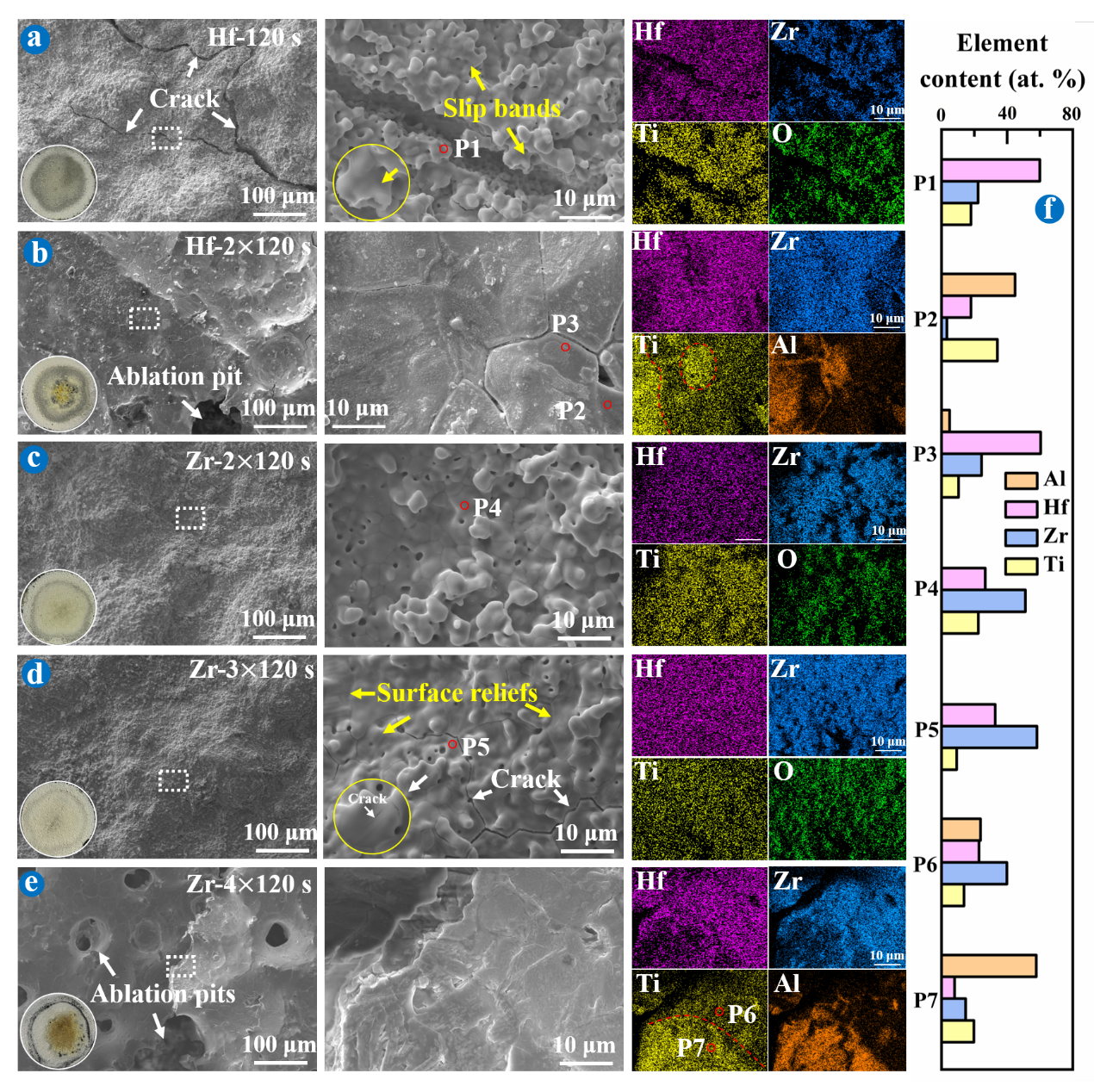


**Figure S3.** Surface compositional and structural attributes of Hf/Zr-MEC-coated C/C composites. a-e) Surface and high magnification SEM images (macroscopic morphologies marked by the inset figures) and corresponding element mappings and f) EDS results of a, b) Hf-MEC-coated and c-e) Zr-MEC-coated samples after repeat ablation with different time: a) 120 s, b, c) 2$\times$120 s, d) 3$\times$120 s and e) 4$\times$120 s.

**Cross-sectional compositional and structural attributes of Hf/Zr-MEC-coated C/C composites.**

Figure S4 shows the cross-sectional structure of the Hf/Zr-MEC-coated samples, which further elucidates their failure behaviors after repeat ablation. After ablation for 120 s, Ti-doped m-(Hf_2/3_Zr_1/3_)O_2_ oxide scales were locally damaged with different directed cracks, including longitudinal penetrating cracks and radial interlaminar cracks, with yellow and pink arrows representing longitudinal and radial cracks in Figures S4a and S4a’, respectively. The growth of radial interlaminar cracks, caused by thermal shock damage, induced a radial penetrating crack for the Hf-MEC coating after ablation for 240 s (Figure S4b). A large amount of detached oxide skeletons indicated complete failure of the outer coating. Compared to the many different directed cracks on the Hf-MEC coating surface, plenty of small pores were dispersed on the Zr-MEC coating surface, after ablation for 240 s (Figures S4c and S4c’). However, these pores provided a channel for oxygen permeation, inducing the formation of a longitudinal penetrating crack after ablation for 360 s (Figures S4d and S4d’). A completely broken oxide skeleton implied the failure of Zr-MEC coating after ablation for 480 s (Figure S4e). As a result, the Zr-MEC coating had superior repeat ablation resistance than the Hf-MEC coating.


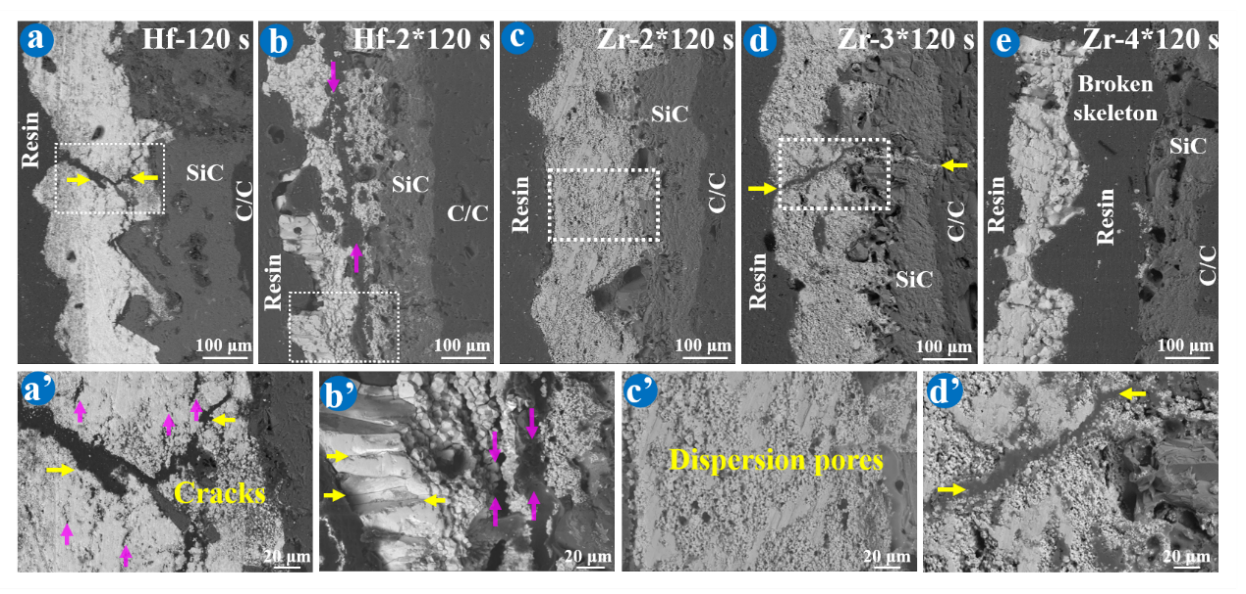


**Figure S4.** Cross-sectional compositional and structural attributes of Hf/Zr-MEC-coated C/C composites. a-e) Cross-sectional BSE images and a’-e’) High magnification images of a-b’) Hf-MEC- and c-e’) Zr-MEC-coated samples after repeat ablation with different times: a, a’) 120 s, b-c’) 2$\times$120 s, d, d’) 3$\times$120 s and e) 4$\times$120 s (the yellow and pink arrows represented the longitudinal and radial cracks, respectively).

**Table S1.** The comparison of oxyacetylene ablation resistance of different UHTC coatings at a heat flux of 2.4 MW/m^2^.

| Types | Line ablation rates (μm/s) | Mass ablation rates (mg/s) | Ablation time | References |
| --- | --- | --- | --- | --- |
| TaC | 1.39 | 2.44 | 30 s | [32] |
| HfC | -0.83 | 0.57 | 60 s | [33] |
| HfC | -0.12 | 0.67 | 60 s | [34] |
| HfC-HfC nanowire | -1.5 | 0.25 | 60 s | [33] |
| HfC | 0.68 | 0.45 | 90 s | [35] |
| HfC | 1.48 | 1.21 | 120 s | [36] |
| HfC | 0.64 | 1.03 | 120 s | [37] |
| HfC | -0.42 | 0.97 | 2*60 s | [33] |
| HfC-HfC nanowire | -0.44 | 0.68 | 120 s | [36] |
| HfC-HfC nanowire | -0.67 | 0.64 | 120 s | [36] |
| HfC-HfC nanowire | -1.53 | 0.41 | 120 s | [36] |
| HfC-HfC nanowire | -0.91 | 0.28 | 2*60 s | [33] |
| HfC | -0.333 | 1.341 | 3*60 s | [33] |
| HfC-HfC nanowire | -0.767 | 0.45 | 3*60 s | [33] |
| ZrC | 1.36 | 0.09 | 90 s | [38] |
| ZrC | 2.25 | 0.06 | 120 s | [39] |
| HfC-ZrC (3:1) | 1.27 | 0.92 | 60 s | [40] |
| HfC-ZrC (1:1) | -0.16 | 0.34 | 60 s | [40] |
| HfC-ZrC (1:3) | 0.14 | 0.57 | 60 s | [40] |
| HfC-ZrC | -0.42 | 1.23 | 120 s | [41] |
| HfC-SiC/HfC | -0.82 | 0.18 | 60 s | [34] |
| SiC/HfC/SiC | 0.12 | 2.5 | 80 s | [42] |
| HfC-SiC (3:1) | 2.16 | 2.68 | 90 s | [43] |
| HfC-SiC (1:1) | 1.08 | 1.2 | 90 s | [43] |
| HfC-SiC (1:3) | 1.63 | 2.34 | 90 s | [43] |
| HfC-SiC | 0.36 | 0.34 | 90 s | [35] |
| MoSi_2_-HfC | -1.626 | -0.488 | 90 s | [44] |
| (SiC/HfC)_3_ | 0.25 | 0.43 | 120 s | [37] |
| HfC-SiC | -0.67 | -0.15 | 120 s | [41] |
| ZrC-SiC | 9.4 | 0.5 | 60 s | [45] |
| (SiC/ZrC)_3_ | 11.3 | 0.75 | 60 s | [45] |
| ZrC-50% SiC | 0.86 | -0.07 | 90 s | [46] |
| ZrC-SiC | 0.08 | 0.29 | 120 s | [39] |
| HfC-TaC | -1.05 | -0.35 | 60 s | [47] |
| TaC/HfC | 0.44 | 0.38 | 60 s | [48] |
| TaC-HfC | -1.104 | -0.253 | 90 s | [44] |
| HfC-TaC | -0.58 | 0.68 | 120 s | [41] |
| HfC-TaC | 0.742 | -0.368 | 120 | [49] |
| HfC-TaC | -1.32 | -0.97 | 120 s | [50] |
| HfC-TaC | 1.83 | 1.88 | 3*60 s | [51] |
| HfC-ZrC-TaC (5:5:1) | -0.694 | -0.231 | 180 s | [9] |
| HfC-ZrC-TaC (3:3:1) | 0.315 | -0.048 | 180 s | [9] |
| Hf-MEC | -1.9 | -1.45 | 60 s | In this work |
| Hf-MEC | -0.93 | -1.21 | 120 s | In this work |
| Hf-MEC | -0.55 | -0.27 | 2*120 s | In this work |
| Zr-MEC | -1.92 | -1.24 | 60 s | In this work |
| Zr-MEC | -0.62 | -0.43 | 120 s | In this work |
| Zr-MEC | -0.49 | -0.25 | 2*120 s | In this work |
| Zr-MEC | -0.32 | -0.15 | 3*120 s | In this work |
| Zr-MEC | -0.11 | -0.03 | 4*120 s | In this work |
